# Ancient and diverged TGF-β signaling components in *Nasonia vitripennis*

**DOI:** 10.1007/s00427-014-0481-0

**Published:** 2014-10-11

**Authors:** Orhan Özüak, Thomas Buchta, Siegfried Roth, Jeremy A. Lynch

**Affiliations:** 1 Institute for Developmental Biology, University of Cologne, Zülpicher Strasse 47b, 50674 Cologne, Germany; 2 Department of Biological Sciences, University of Illinois at Chicago, 900 S. Ashland Ave, Chicago, IL 60607 USA

**Keywords:** Dorsoventral patterning, BMP, Activin, RNA localization, Oogenesis

## Abstract

The transforming growth factor beta (TGF)-β signaling pathway and its modulators are involved in many aspects of cellular growth and differentiation in all metazoa. Although most of the core components of the pathway are highly conserved, many lineage-specific adaptations have been observed including changes regarding paralog number, presence and absence of modulators, and functional relevance for particular processes. In the parasitic jewel wasp *Nasonia vitripennis*, the bone morphogenetic proteins (BMPs), one of the major subgroups of the TGF-β superfamily, play a more fundamental role in dorsoventral (DV) patterning than in all other insects studied so far. However, *Nasonia* lacks the BMP antagonist Short gastrulation (Sog)/chordin, which is essential for polarizing the BMP gradient along the DV axis in most bilaterian animals. Here, we present a broad survey of TGF-β signaling in *Nasonia* with the aim to detect other lineage-specific peculiarities and to identify potential mechanisms, which explain how BMP-dependent DV pattering occurs in the early *Nasonia* embryo in the absence of Sog.

**Electronic supplementary material:**

The online version of this article (doi:10.1007/s00427-014-0481-0) contains supplementary material, which is available to authorized users.

## Introduction

The transforming growth factor beta (TGF-β) signaling pathway controls various processes throughout development, which include dorsoventral axis specification, cell proliferation, appendage formation, as well as the central nervous system patterning (Chang et al. 2002; Parker et al. 2004). It consists of the extracellular ligands and their modulators, which interact with each other to control ligand availability and distribution, of the receptors and of intracellular components, which transduce the signal to the nucleus and regulate target gene expression (Parker et al. 2004; Sieber et al. 2009) (Table 1).

**Table 1:** BMP pathway components of *Nasonia* and their homologs in *Drosophila* and *Tribolium*

	*Nasonia vitripennis*		*Drosophila melanogaster*	*Tribolium castaneum*
BMP	NvDpp XP_001607677.1	1, 2	DmDpp AAN10431.1	TcDpp EFA02913.1
	NvGbb1 XP_001603876.1	1, 2	DmGbb AAF47075.1	TcGbb1 EFA04645.1
	NvGbb2 XP_001603269.2	1, 2	DmScw AAA56872	TcGbb2 EFA04646.1
	NvADMP XP_001604750.2	2	–	–
Maverick	NvMav XP_001606148.2	2	DmMav AAF59328.1	TcMav EFA11885.1
Activin	NvAct XP_001602284.1	2	DmAct NP_651942.2	TcAct EFA05602.1
	NvDaw (Apl) XP_003425497.1	2	DmDaw NP_523461.1	TcAlp EFA11884.1
	NvMyo XP_001602255.2	2	DmMyo AAF59319.1	TcMyo EFA05753.1
Type I receptor	NvTkv XP_001601240.2	2	DmTkv AAN10533	TcTkv EFA09250.1
	NvSax XP_003426889.1	2	DmSax AAF59189	TcSax EFA07576.1
	NvBaboon XP_003427942.1	2	DmBaboon AAF59011	TcBaboon EFA01312.1
Type II receptor	NvPunt1 XP_001606053.1	2	DmPut AAF55079	TcPut EEZ97734.1
	NvPunt2 XP_001603863.1	2	–	–
	NvWit XP_003428148.1	2	DmWit AAF47832	TcWit XP_974821.1
R-Smads	NvMad1 XP_001601460.2	2	DmMad AAF51142.1	TcMad EFA05663.1
	NvMad2 XP_001602991.1	2	–	–
	NvSmox XP_001608214.2	2	DmSmox NP_511079.1	–
Co-Smads	NvMedea XP_003427724.1	2	DmMedea NP_524610.1	TcMedea EFA11586.1
Cross-veinless 2	NvCv2a XP_001601040.2	2	DmCv2 AAG01337.2	TcCv2 EFA10783.1
Cross-veinless 1	NvCv2b XP_001599339.1	2	–	–
	NvCv2c XP_001603432.2	2	–	–
	NvCv2d XP_001599102.2	2	–	–
	NvTsg1 XP_001600362.1	1, 3	DmTsg NP_511135.1	TcCv1 XM_001812724.2
	NvTsg2 XP_001603630.2	3	DmCv1 AHN59374	–
	NvTsg3 XP_001607991.2	3	DmSrw AAF47878.2	–
Tolloid	Nvtolloid XP_008211528.1	1	DmTld AAF56329.2	TcTld XP_970162.1
	–		DmTok AHN57509.1	–
Follistatin	NvFollistatin XP_001607105.2	2	DmFs AAF58158	TcFs EFA07639
Extracellular modulators	NvDally XP_003425386.1	2	DmDally AAA97401	TcDally XP_008194004
	NvGlypican4 XP_001607767.2	2	DmDlp NP_524071	TcGlypican6 XP_974946.2
	NvCollagenIV XP_003427100.1	2	DmCollIV AAF52204	TcCollIV XP_008193734
	NvPentagone XP_001601094.2	2	DmPent AAM71054	TcPent XP_967285

The NCBI accession number is given with the gene name. The numbers to the right of the *Nasonia* genes (gray column) indicate sources for phylogenetic analysis, expression studies, and/or functional (RNAi) data. 1, Özüak et al. 2014; 2, this study; 3, Nunes da Fonseca et al. 2010

Most of the ligands belong to either the bone morphogenetic protein (BMP) subfamily or to the Activin/TGF-β subfamily (Yamamoto and Oelgeschläger 2004; Hinck 2012). Around 30 TGF-β ligands have been described in vertebrates, while *Drosophila* and *Tribolium* each have seven (Schmierer and Hill 2007; Van der Zee et al. 2008). Decapentaplegic (Dpp), Glass bottom boat (Gbb), and Screw (Scw) are the BMP-like ligands in *Drosophila* (Padgett et al. 1987; Wharton et al. 1991; Arora et al. 1994), while Activin (Act), Activin-like protein 23b, which is also called Dawdle (Daw), and Myoglianin (Myo) belong to the Activin/TGF-β subfamily (Kutty et al. 1998; Lo and Frasch 1999; Parker et al. 2006). Maverick (Mav) is highly diverged and is not easily placed into either of the ligand subfamilies (Nguyen et al. 2000).

All TGF-β ligands form dimers and bind to a heteromeric receptor complex of two type I and two type II serine-threonine kinase receptors (Sieber et al. 2009). In *Drosophila*, the BMPs as well as the Activins use Punt as a common type II receptor, while specificity is generated by the type I receptors. BMP ligands bind the type I receptors Thickveins (Tkv) and Saxophone (Sax), whereas Activin/TGF-β ligands signal through the type I receptor Baboon (Babo) (Parker et al. 2004).

Upon ligand binding, the type I receptors become phosphorylated, which in turn phosphorylate and thereby activate receptor-regulated SMADs (R-SMADs). Tkv and Sax activate Mothers against Dpp (Mad), and Babo activates SMAD on X (Smox). Once phosphorylated, R-SMADs bind to the Co-SMAD Medea and form a complex that translocates into the nucleus and regulates target gene expression (Parker et al. 2004).

At the extracellular level, a large variety of modulators are involved in controlling ligand availability and distribution. A prominent group of modulators, which usually act as BMP antagonists are characterized by an array of conserved cysteine-rich domains that form cysteine knot structures (Walsh et al. 2010). On the basis of spacing of the cysteine residues within the cysteine ring, they have been grouped into several subgroups, e.g., the Noggins, the Chordin family, the Twisted gastrulation-like proteins (Crossveinless 1), the Crossveinless 2, and the Dan family (Walsh et al. 2010). In *Drosophila*, the main BMP antagonist in dorsoventral patterning is the Chordin homolog Short gastrulation (Sog), which interacts with Twisted gastrulation and is cleaved by the metalloprotease Tolloid (O’Connor et al. 2006).

Besides diffusible, secreted proteins, several membrane-bound and extracellular matrix proteins have been shown to influence the efficiency of TGF-β signaling (Ramel and Hill 2012). Thus, in the *Drosophila* wing, two glypicans, the GPI anchored heparan-sulfate proteoglycans Dally and Dally-like, are required for efficient BMP signaling activity (Erickson 2011) while type IV collagens control the range of the BMP signaling gradient in the embryo (Ashe 2008).

In our previous work, we showed that the parasitic jewel wasp *Nasonia vitripennis* uses the BMP pathway to pattern the dorsoventral (DV) axis despite the fact that no *sog* ortholog is present in the *Nasonia* genome (Özüak et al. 2014). The main goals of this work are to provide an overview of the TGF-β pathway in *Nasonia* and to identify components which might help to explain how the BMP signaling gradient is established during DV patterning in the wasp embryo.

Similar work was already done for the short germ beetle *Tribolium castaneum*, which revealed that the beetle retained a more ancestral complement of TGF-β signaling components compared to *Drosophila* (van der Zee et al. 2006; Van der Zee et al. 2008; Nunes da Fonseca et al. 2010).

Here, we comprehensively identify and describe components of the TGF-β signaling pathway in *Nasonia*. Interestingly, we found a case of parallel evolution, involving the duplication and divergence of the BMP 5/7 ligands in *Nasonia* and *Drosophila.* In addition, we identified the BMP ligand ADMP, which is not present in *Drosophila* and *Tribolium*, but plays an important role in vertebrates (Reversade and De Robertis 2005). As we were unable to find a *sog* homolog in *Nasonia*, we were especially interested to identify alternative BMP antagonists, which might be expressed at the ventral side of the embryo. However, we failed to identify such inhibitors corroborating our functional studies which indicated that the DV BMP gradient of *Nasonia* is not shaped by an opposing inhibitory gradient, but rather by diffusion from a dorsal source region (Özüak et al. 2014). Interestingly, the RNA of the type I receptor Tkv is localized to the dorsal midline of the developing *Nasonia* oocyte. In addition, one of the type II receptors is dorsally expressed in the early embryo. Based on these observations, we discuss a possible scenario of how the embryonic BMP gradient in *Nasonia* is established in the absence of a ventral inhibitor.

## Material and methods

### Embryo and ovary collection

All *N. vitripennis* embryos were collected using the *Waspinator* and fixed as described by (Buchta et al. 2013).

Nasonia ovaries were dissected and fixed as described by (Lynch et al. 2010a).

### ISH

Single- and two-color in situ hybridizations were performed as previously described (Brent et al. 2003; Lynch et al. 2010a). For list of primer used to produce probes, see Electronic supplementary material (ISH primer).

### RNAi

Young *N. vitripennis* pupae were injected as previously described (Lynch and Desplan 2006). In average, 25–35 females were injected and 40–75 embryos of the appropriate ages were analyzed. Most genes tested here showed zygotic expression. After parental RNA interference (pRNAi), neither female sterility nor lethality was observed. Maternally expressed genes showed either sterility (*Nv-punt2*, *Nv-smox*) or embryonic lethality (*Nv-mad2*) with 100 % penetrance after pRNAi .

### Identification and phylogenetic analyses of *Nasonia* orthologs of BMP pathway components

Orthologs were identified by reciprocal best BLAST hits of the *Drosophila* genes of interest to the *Nasonia* genome or transcriptome assemblies (Werren et al. 2010).

Multiple alignments were carried out using ClustalW (http://www.ebi.ac.uk/clastalw), and maximum likelihood phylogenies were generates with MEGA version 5 (Tamura et al. 2011)

## Results

### Ligands

**Fig. 1:**
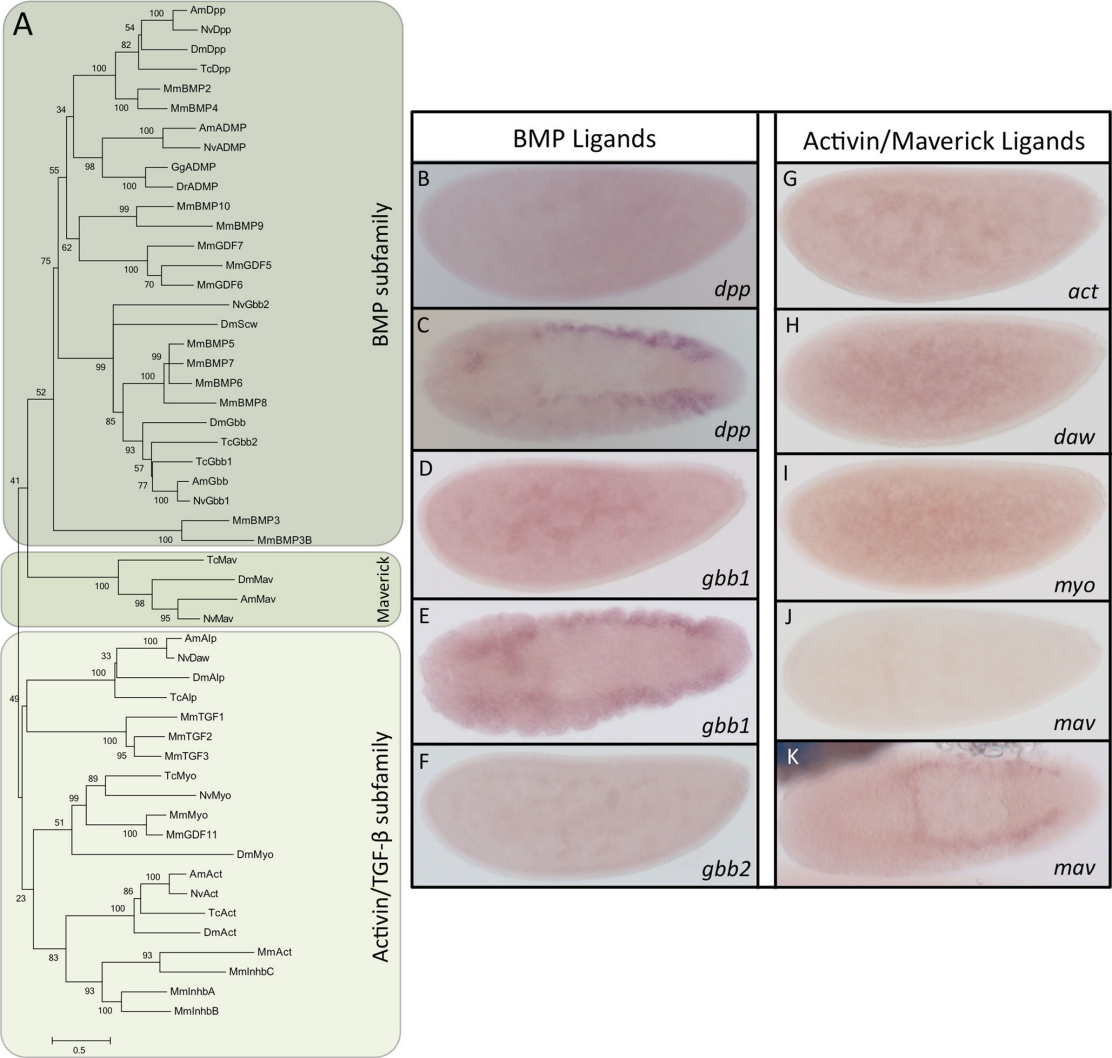
Ligands. **a** Maximum likelihood tree of TGF-β ligands in different insect and vertebrate species. Bootstrap values (1,000 replicates) are indicated in percentages. Amino acid substitution model: WAG + i + g. *Nv Nasonia vitripennis*, *Am Apis mellifera*, *Dm Drosophila melanogaster*, *Tc Tribolium castaneum*, *Mm Mus musculus*, *Gg Gallus gallus*, *Dr Danio rerio.* **b**, **d**, **f**, **g**, **h**, **i**, **j** Early and **c**, **e**, **k** late ISH of BMP ligands (**b**–**f**) and Activin/Maverick ligands (**g**–**k**) for *dpp* (**b**, **c**), *gbb1* (**d**, **e**), *gbb2* (**f**), *act* (**g**), *daw* (**h**), *myo* (**i**), and *mav* (**j**, **k**). **b**, **d**, **f**, **g**, **h**, **i**, **j** Lateral view. **c**, **e**, **k** Dorsal view. Anterior is left (accession numbers are given in Table 1 and Electronic supplementary material)

**Fig. 2:**
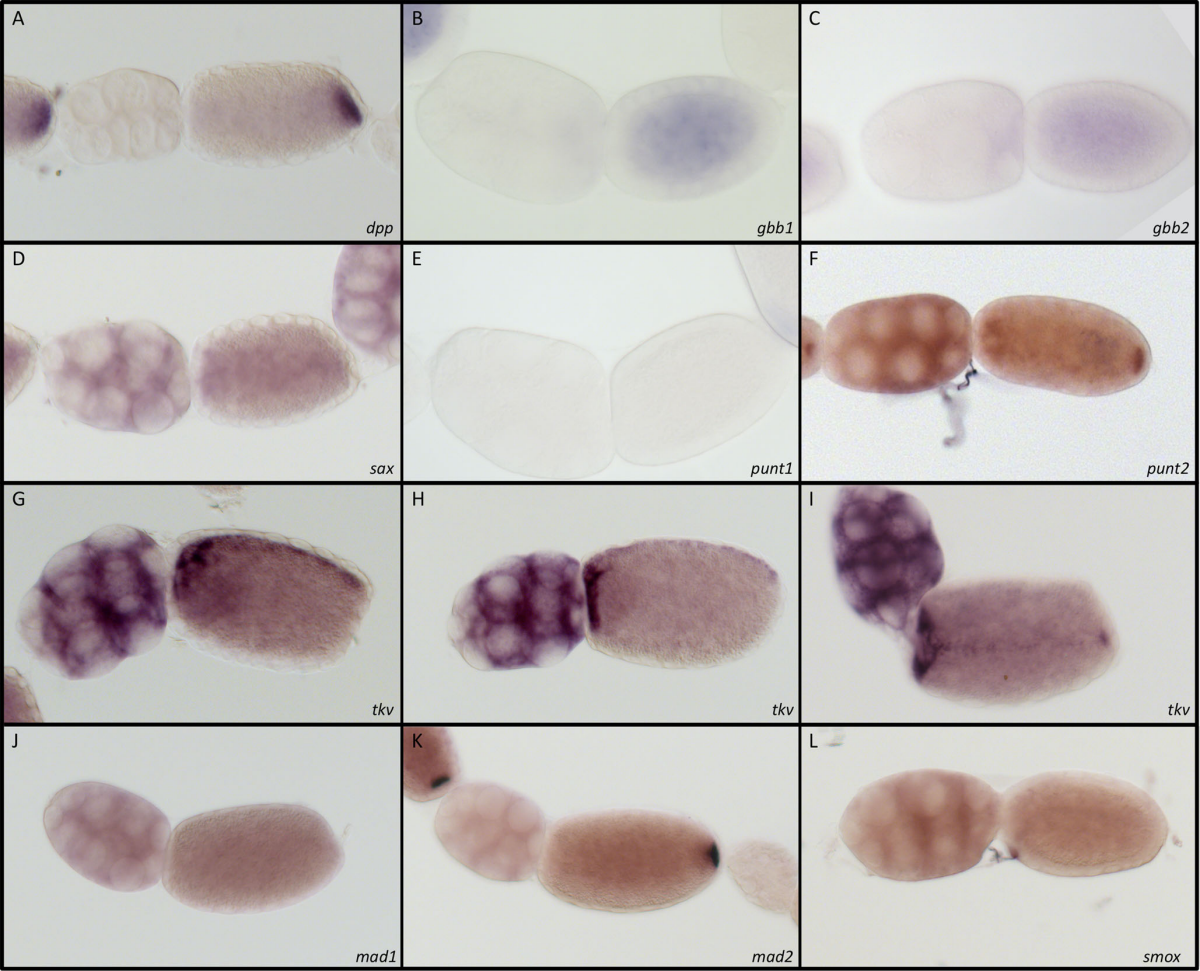
BMP components in *Nasonia* ovaries. Expression of **a** *dpp*, **b** *gbb1*, **c** *gbb2*, **d** *sax*, **e** *punt2*, **f** *punt1*, and **g**–**i** *tkv* in lateral (**g**–**h**) and dorsal (**i**) views, *mad1* (**j**), *mad2* (**k**), and *smox* (**l**) in *Nasonia* ovaries (accession numbers are given in Table 1 and Electronic supplementary material)

So far, seven TGF-β ligands are described in *Drosophila* and *Tribolium*. In the wasp *Nasonia*, we found eight potential TGF-β ligands in the genome. In our previous work, we described the important role of *Nv-dpp* in patterning the dorsal-ventral axis of the wasp embryo (Özüak et al. 2014). Knocking down *Nv-dpp* leads to the loss of dorsal fates and an almost complete ventralization of the embryo. In our phylogenetic analysis, *Nv-dpp* groups perfectly with other insect Dpp sequences, as well as their vertebrate homologs Bmp2/4 (Fig. 1a). During oogenesis, *Nv-dpp* is expressed in the nurse cells and localized at the posterior pole of the oocyte (Fig. 2a). In the early embryo, *Nv-dpp* is ubiquitously expressed and after gastrulation is completed, it is expressed in two stripes that flank the extraembryonic region on the dorsal side of the embryo (Fig. 1b, c).

Another important ligand for embryonic DV patterning in *Drosophila* is Screw, which represents a diverged paralog of Gbb (Wharton et al. 1991; Arora et al. 1994). *Drosophila* Gbb plays no role in DV patterning but is required in later developmental processes such as wing formation (O’Connor et al. 2006). We found two paralogs of Nv-Gbb, but unlike in *Tribolium*, where both Gbb paralogs are closer related to each other than to any other Gbb (Van der Zee et al. 2008), the *Nasonia* Gbb paralogs are not closely related. Instead, Nv-Gbb1 groups together with other insect Gbbs and the vertebrate homologs BMP5/7, while Nv-Gbb2 shows signs of a very fast evolving gene such as *Drosophila screw*. Both genes are ubiquitously expressed during oogenesis as well as during early embryogenesis (Fig. 1d, f; Fig. 2b, c). After gastrulation *Nv-gbb1* is expressed like *Nv-dpp* in two stripes at the dorsal side (Fig. 1e), while *Nv-gbb2* expression is gone. The knockdown of Nv-Gbb2 leads, like the knockdown of Nv-Dpp to a ventralization of the embryo (Özüak et al. 2014). Interestingly, the knockdown of Nv-Gbb1 has no embryonic phenotype, suggesting that it plays no role in early embryonic DV patterning. It might however play a role in later embryonic or in postembryonic stages, which we could not target by parental RNAi. Thus, the situation might be similar to *Drosophila* where one Bmp5/7 paralog (*screw*) was subject to fast evolutionary changes adapting to the special requirements of early embryonic DV patterning while the other one (*gbb*) retaining ancestral sequence features is required for later (more generic) developmental processes.

Anti-dorsalizing morphogenetic protein (ADMP) has a BMP-like activity in *Xenopus* (Reversade and De Robertis 2005). Despite its important role in vertebrates during DV patterning, ADMP is not present in either *Drosophila* or *Tribolium*. However, it is present in the honeybee and we found an ortholog of ADMP in the wasp (Fig. 1a). It will be interesting to further investigate ADMP function in *Nasonia* to see if it also plays a role in self-regulation during embryonic DV patterning.

Orthologs of ligands belonging to the Activin subfamily such as *act*, *daw*, and *myo* are found in *Nasonia*. All three are ubiquitously expressed in the early embryo and later no expression is detectable (Fig. 1g–i). The last of the eight detected ligands is Maverick, which is not clearly grouped into one of the two large subfamilies. In *Drosophila*, Mav is required for growth in the wing disc. A knockdown of Mav leads to wing size reduction with normal vein patterns (Hevia and de Celis 2013). We investigated the expression pattern of *Nv-mav* and found that there is no expression in the early embryo, but later after completion of gastrulation, *Nv-mav* is expressed in two very narrow stripes flanking the extraembryonic region on the dorsal side (Fig. 1j, k).

### Receptors

**Fig. 3:**
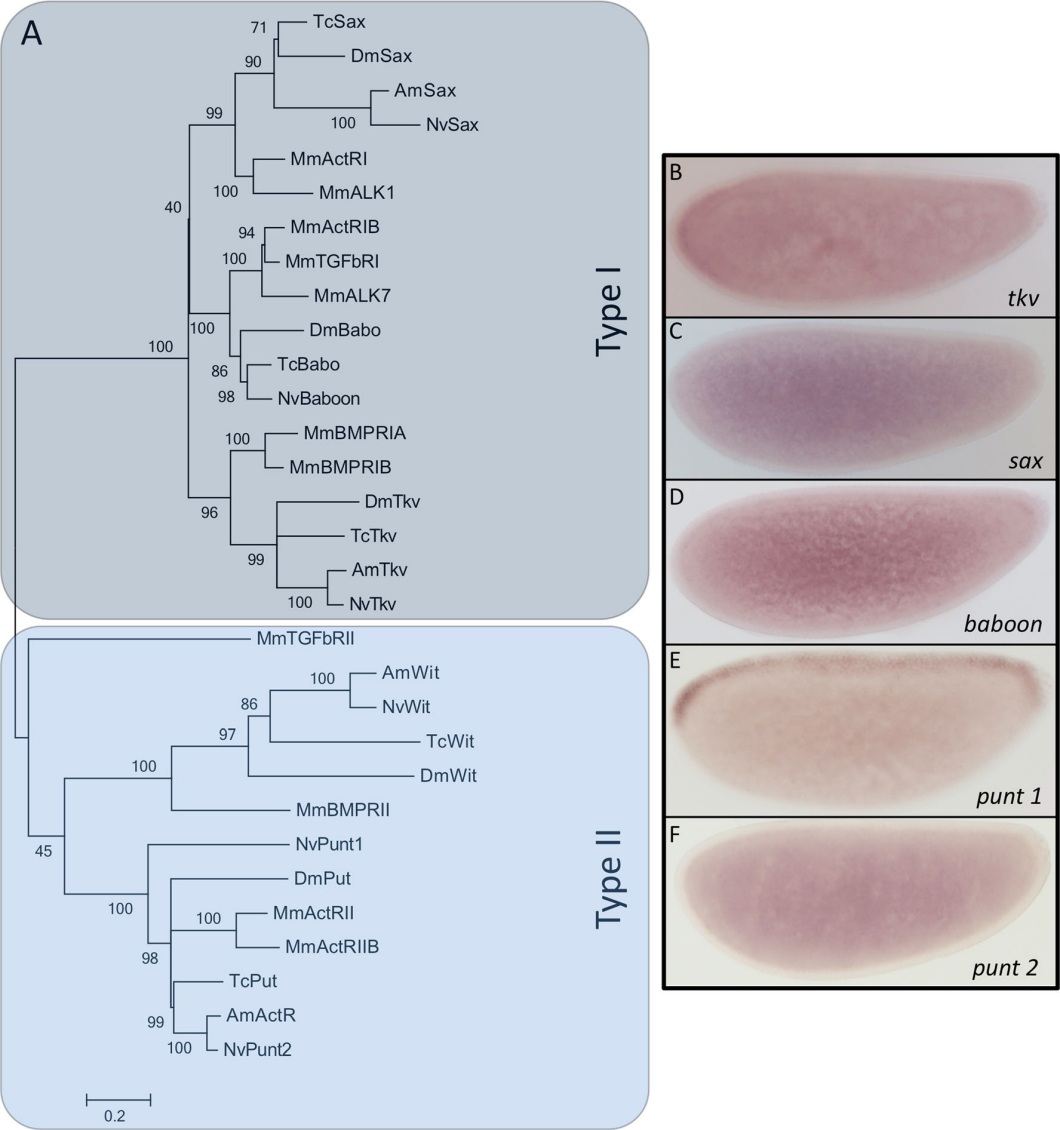
Receptors. **a** Maximum likelihood tree of type I and type II receptors in different insect and vertebrate species. Bootstrap values (1,000 replicates) in percentages. Amino acid substitution model: WAG + i + g. *Nv Nasonia vitripennis*, *Am Apis mellifera*, *Dm Drosophila melanogaster*, *Tc Tribolium castaneum*, *Mm Mus musculus*; see main text for protein abbreviations. Expression patterns of **b** *thickveins*, **c** *saxophone*, **d** *baboon*, **e** *punt1*, and **f** *punt2* in *Nasonia* embryos. Anterior is left (accession numbers are given in Table 1 and Electronic supplementary material)

The extracellular BMP signal is transmitted via tetrameric receptor complexes formed by type I and type II receptors. Altogether, our analyses revealed six *Nasonia* orthologs of *Drosophila* serine-threonine kinase receptors: two BMP type I receptors, *tkv* and *sax*, and the Activin receptor type I ortholog *babo*, as well as type II receptors *wishful thinking (wit)* and two *punt* orthologs (Fig. 3a). Aside from these receptors, we also found BAMBI (not shown), which in vertebrates is known to be a pseudo-receptor (Onichtchouk et al. 1999) and might play a role in inhibiting BMP signaling in *Nasonia* as well.

*Nv-sax* is ubiquitously expressed in the nurse cells and ubiquitously distributed in the oocyte (Fig. 2d). *Nv-tkv* is also expressed in all nurse cells. However, in the oocyte, it shows a striking pattern of localization. Besides an anterior accumulation, the RNA is found in a narrow stripe along the dorsal midline (Fig. 2g–i). The dorsal side can be identified via the position of the oocyte nucleus. (data not shown, (Lynch et al. 2010b)). Thus, *Nv-tkv* RNA localization during oogenesis closely resembles that of the *Nv-tgf-α*, which is involved in establishing the DV axis in *Nasonia* (Lynch et al. 2010b). In early embryos, *Nv-tkv* is no longer localized and shows like *Nv-sax* ubiquitous expression (Fig. 3b, c). Knockdown of both receptors using pRNAi has been performed in previous studies and resulted in the case of *Nv-sax*, as well as *Nv-tkv* in a strong ventralization of the embryo (Özüak et al. 2014).

Similar to the type I receptors, *Nv-babo* shows a uniform expression pattern in early *Nasonia* embryos (Fig. 3d). The presence of a *babo* homolog in *Nasonia* is interesting since previous studies on *Apis mellifera* revealed a lack of this gene (Van der Zee et al. 2008), indicating that the loss of Activin signaling via Baboon is not a general feature of the hymenoptera.

Furthermore, two homologous genes of the type II receptor Punt are present. *punt1* is not expressed during oogenesis (Fig. 2e). However, it shows a distinct expression pattern along the dorsal midline in early blastoderm embryos (Fig. 3e), which strongly resembles the early expression patterns of previously described BMP signaling target genes in *Nasonia* (Buchta et al. 2013).

In contrast to this, *punt2* is expressed during oogenesis and is localized at the posterior pole of the oocyte (Fig. 2f). This localization is not present in the embryo where *punt2* messenger RNA (mRNA) is distributed ubiquitously (Fig. 3f). Because of its maternal distribution, we knocked down *punt2*. Enclosed females that were injected with *punt2* double-stranded RNA (dsRNA) during yellow pupae stage laid no eggs and had degenerated ovaries (data not shown). This phenotype could be due to defects in Activin or BMP signaling as *punt2* might be essential for both pathways*.*

### SMADs

**Fig. 4:**
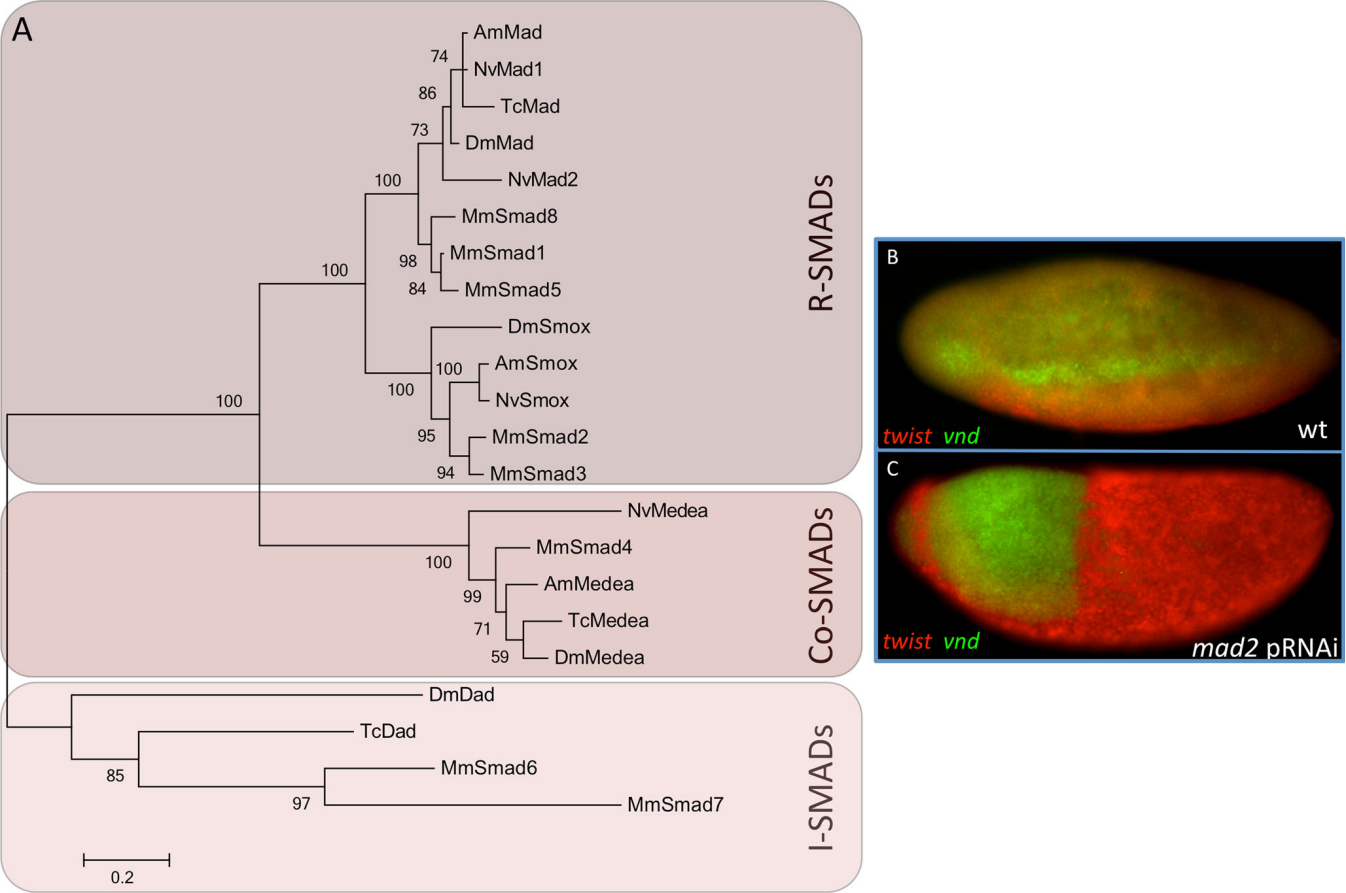
SMADs. **a** Maximum likelihood tree of SMADs in different insect and vertebrate species. Bootstrap values (1,000 replicates) in percentages. Amino acid substitution model: WAG + i + g. *Nv Nasonia vitripennis*, *Am Apis mellifera*, *Dm Drosophila melanogaster*, *Tc Tribolium castaneum*, *Mm Mus musculus*. Lateral view of wild-type (**b**) and *mad2* knockdown (**c**) *Nasonia* embryos showing expression pattern of *twi* (*red*) and *vnd* (*green*). Anterior is left (accession numbers are given in Table 1 and Electronic supplementary material)

SMADs are characterized by the presence of two Mad homology (MH1 and MH2) domains. In addition, R-SMADs have a C-terminal SXS motif. Upon ligand binding, the receptors pass on the signal by phosphorylating both serines in the SXS motif thereby activating the R-SMADs (Feng and Derynck 2005). We identified four proteins with a MH1 and MH2 domain in the *Nasonia* genome, three of which contained a SXS motif (Fig. S1). Phylogenetic analysis revealed that two of the SXS motif containing proteins group together with *Drosophila* Mad and the third one with *Drosophila* Smox (Fig. 4a). It is worth mentioning that Nv-Mad2 is, similar to Nv-Gbb2 and Nv-Punt1, significantly more diverged in comparison to Nv-Mad1 and the other insect Mad orthologs. The fourth identified protein displayed clear homology to the Co-SMAD Medea (Fig. 4a). We could not identify any orthologs of known inhibitory SMADs like Daughters against dpp (Dad) (Tsuneizumi et al. 1997).

During oogenesis, *Nv-mad1* is ubiquitously expressed in the nurse cells and oocytes while *Nv-mad2* RNA accumulates at the posterior pole of the oocyte (Fig. 2j, k). *Nv-mad1* does not seem to have a conserved role in embryogenesis since it did not show any phenotype after pRNAi mediated knockdown. However, embryos of females injected with *Nv-mad2* dsRNA were severely ventralized as seen from the expansion of the ventral *twist* domain (Fig. 4c). This phenotype closely resembles the previously described knockdown phenotype of *Nv-dpp* and *Nv-gbb2* (Özüak et al. 2014).

*Nv-smox* is ubiquitously expressed during oogenesis (Fig. 2l), and pRNAi-mediated knockdown resulted in sterile females (not shown) indicating an important role of Activin signaling during oogenesis. The *Nv-smox* phenotype suggests that the sterility caused by *Nv-punt2* knockdown is due to interference with Activin, rather than BMP signaling.

### TGF-β modulators

**Fig. 5:**
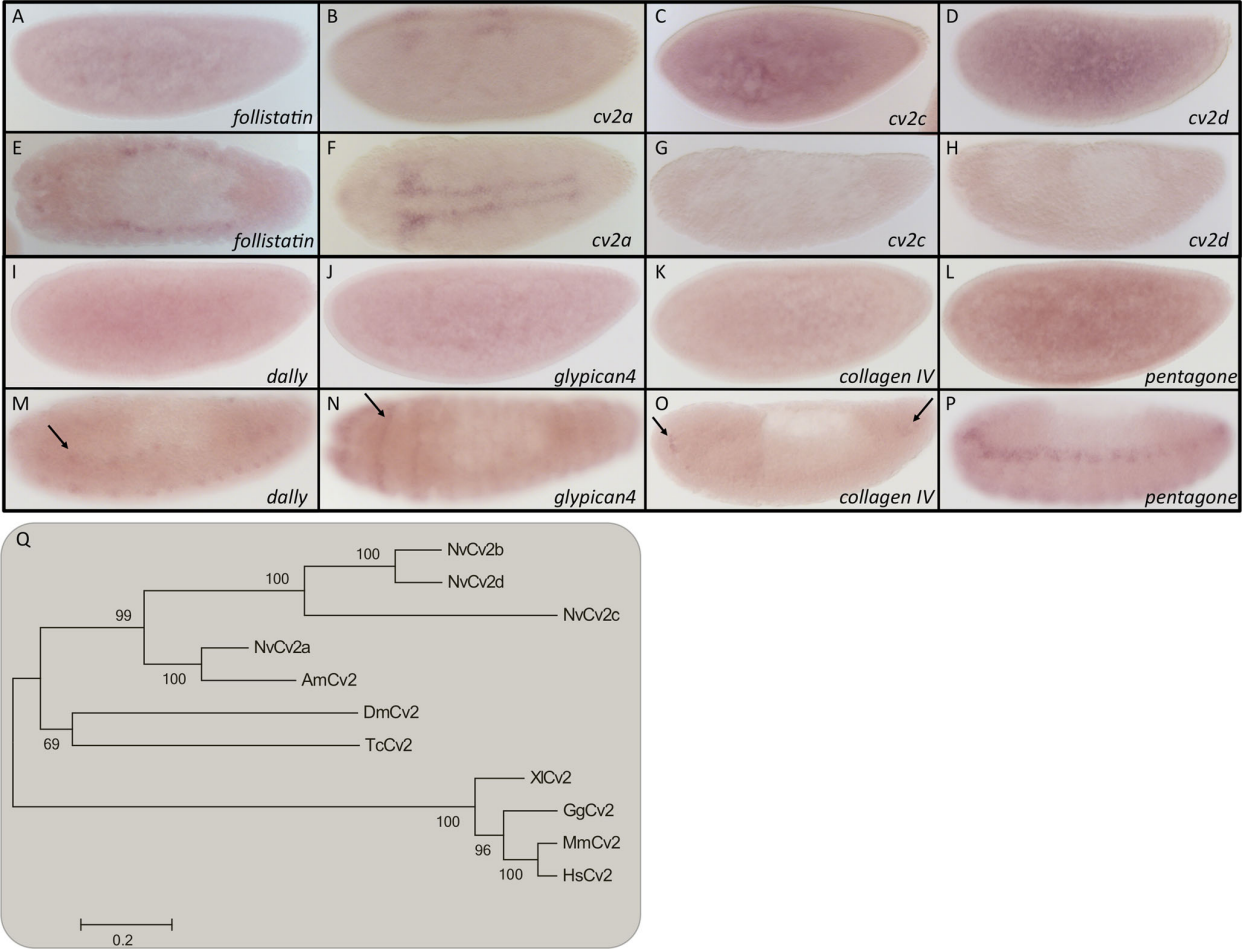
Extracellular modulators. Early (**a**–**d**, **i**–**l**) and late (**e**–**h**, **m**–**p**) expression of *follistatin* (**a**, **e**), *cv2a* (**b**, **f**), *cv2c* (**c**, **g**), *cv2d* (**d**, **h**), *dally* (**i**, **m**), *glypican4* (**j**, **n**), *collagen IV* (**k**, **o**), *pentagone* (**l**, **p**) in *Nasonia* embryos. **a**–**d**, **i**–**l**, **o**, **p** Lateral view. **e**, **f** Dorsal view. **g**, **h**, **o** Dorsolateral view. **n** Ventral view. Anterior is the right. **q** Maximum likelihood tree of *crossveinless2* in different insect and vertebrate species. Bootstrap values (1,000 replicates) in percentages. Amino acid substitution model: WAG + i + g. *Nv Nasonia vitripennis*, *Am Apis mellifera*, *Dm Drosophila melanogaster*, *Tc Tribolium castaneum*, *Mm Mus musculus*, *Hs Homo sapiens*, *Gg Gallus gallus*, *Xl Xenopus laevis* (accession numbers are given in Table 1 and Electronic supplementary material)

At the extracellular level, the TGF-β pathway is influenced in many ways to fine tune ligand and receptor activity. While antagonists bind to ligands and inhibit receptor interactions, cell surface proteins modulate the flux of the ligands (Ramel and Hill 2012). In our previous work, we provided evidence that *Nasonia* is lacking *sog*, which is the main BMP antagonist in *Drosophila* and other insects (Buchta et al. 2013). Sog belongs to the Chordin family, one of the subgroups of BMP antagonists. We searched for orthologs of other groups such as Noggin or members of the Dan family. While in *Tribolium*, two members of the Dan family, Dan and Gremlin, are present, no orthologs of members of the Dan family nor Noggin could be identified in the *Nasonia* genome using searches based on reciprocal best BLAST hits. However, like in *Drosophila* and *Tribolium*, we found an ortholog of Follistatin, which preferentially antagonizes Activin rather than BMP (Pentek et al. 2009). In *Nasonia*, *follistatin* is ubiquitously expressed during early embryogenesis and later after gastrulation, it is expressed in two narrow stripes that flank the extraembryonic region on the dorsal side of the embryo (Fig. 5a, e)

Finally, we were able to identify four Crossveinless 2 like molecules (Cv2a- Cv2d) (Fig. 5q). Cv2 proteins consist of similar domains as Sog/chordin proteins, but are thought to tightly interact with the type I receptors and/or membrane anchored proteoglycans and therefore lack diffusibility (Ambrosio et al. 2008). *cv2c* and *cv2d* show a uniform expression in early *Nasonia* blastoderm embryos and weak uniform expression during later embryonic development (Fig. 5c, d, g, h). *cv2a* is first expressed in small patches on the dorsal side at the cellularized blastoderm stage (Fig. 5b). During development, these patches widen toward the anterior and elongate toward posterior. At the onset of gastrulation, *cv2A* is expressed in two narrow stripes flanking the presumptive serosa (Fig. 5f). We were not able to amplify *cv2b* from ovarian or embryonic cDNA, indicating that this gene might be a pseudogene or is expressed only during postembryonic development.

Besides the antagonists, we found several orthologs of cell surface proteins that are candidates for modulating the flux of BMP ligands and facilitating long-range signaling activity. Among these are orthologs of Dally, Glypican4, Collagen IV, and Pentagone (Vuilleumier et al. 2010; Ramel and Hill 2012). In early blastoderm embryos, all are ubiquitously expressed (Fig. 5i–l). Later, after gastrulation is completed, all of them show a distinct pattern. *Nv-dally* is expressed in a dotted pattern of two stripes flanking the ventral midline (Fig. 5m). *Nv-glypican4* is expressed in segmental stripes on the ventral half of the embryo (Fig. 5n). *Nv-collagen IV* is expressed in small patches at the anterior and posterior of the embryo (Fig. 5o). The post-gastrulation expression pattern of *Nv-pentagone* in two ventral stripes resembles strongly that of *Nv-ind*, a neuroectodermal marker, which we described in our previous work (Fig. 5p) (Buchta et al. 2013).

## Discussion

Taken together, this work indicates that *Nasonia* has retained some components of an ancestral TGF-β signaling network, such as ADMP and BAMBI, that have been lost in other insect lineages (Van der Zee et al. 2008). However, *Nasonia* also shows high degrees of divergence in some key signaling components with essential functions for embryonic DV patterning like Gbb2, Mad2, and Punt1. The most striking observation is that *Nasonia* apparently has lost some families of BMP antagonists including Sog which plays a crucial in DV pattering in most bilaterian animals (Mizutani and Bier 2008).

Although the inability to identify a gene within genome and transcriptome data represents only negative evidence, there are several observations supporting the claim that the BMP system in *Nasonia* operates without Sog: (1) Sog cannot be found in the closely related wasp *Trichogramma* (JAL personal observation); (2) In *Apis*, a *sog* gene has been identified, but it appears to lack expression in early blastoderm embryos when DV patterning is expected to take place (Wilson et al. 2014); and (3) Nv-Tolloid, the metalloprotease universally required to cleave Sog within Sog-BMP complexes in order to release active BMP, has only restricted anterior expression, and pRNAi does not result in DV pattering defects (Özüak et al. 2014).

Despite the absence of *sog*, three *Nasonia* orthologs of Twisted gastrulation/Crossveinless1 (Nv-CvA-CvC) were discovered in an earlier study (Nunes da Fonseca et al. 2010). In *Drosophila*, Tsg is thought to be a part of the Sog–Dpp complex. In this complex, Tsg can have a pro-BMP as well as an anti-BMP function. It was suggested that Tsg acts anti-BMP by enhancing the binding of Sog to Dpp and pro-BMP by enhancing the cleavage of Sog by the metalloprotease Tolloid (O’Connor et al. 2006). In our previous studies with *Nasonia* using *Nv-tsg1* pRNAi, a pro-BMP role for Tsg during embryogenesis was observed since embryos of injected females were severely ventralized (Özüak et al. 2014).

However, our inability to identify *sog*, as well as the lack of DV patterning function of Tolloid, suggest that the pro-BMP function of Nv-Tsg is Sog-independent. An exclusively pro-BMP role for Tsg was also observed in *Tribolium castaneum* (Nunes da Fonseca et al. 2010). Interestingly, this pro-BMP function of Tc-Tsg could be shown to be Sog independent, too. Thus, both in *Tribolium* and *Nasonia*, Tsg plays a major, albeit Sog-independent role in DV patterning. In *Drosophila*, Tsg combines Sog-dependent and Sog-independent functions (Wang and Ferguson 2005) while in a more basal insect, we recently observed that Tsg predominantly acts in Sog-dependent manner (unpublished observations). Together, these findings illustrate that the interactions of extracellular BMP modulators are subject to considerable evolutionary changes which probably reflect adaptations to the ways by which early BMP gradients from in the embryo.

The most important observation supporting the absence of a ventral BMP inhibitor like Sog in *Nasonia* is the phenotype caused by loss of Toll signaling (Özüak et al. 2014). In *Nasonia*, Toll is required to establish ventral-most cell fates, in particular, the mesoderm. However, Toll is not involved in patterning the dorsal half. Loss of function analysis shows that dorsal patterning depends on a BMP signaling gradient which apparently is establish in the absence of Toll, i.e., in the absence of an inhibitor which is ventrally activated by Toll signaling. As in all other insects studied so far, Sog is one of the key target genes activated by Toll signaling at the ventral side, this phenotypic analysis strongly corroborates our claim that the BMP gradient in *Nasonia* is not established through the formation an opposing inhibitor gradient.

Thus, *Nasonia* apparently establishes its DV axis in a bipolar fashion using independent signaling sources along the ventral and dorsal midline (Özüak et al. 2014). These signaling sources are likely to be established already during oogenesis. The oocytes of *Nasonia* like those of *Apis*, the other hymenopteran species studied so far, show an amazing ability to precisely localize mRNAs not only to the anterior and posterior poles (Fig. 2a, g) but also along the dorsal midline (Lynch et al. 2006, 2010b; Wilson and Dearden 2013). Thus, in *Nasonia* and *Apis*, the RNA of TGF-α is localized in a narrow dorsal stripe and the secreted TGF-α ligand initiates EGF signaling at the dorsal side of the follicular epithelium along the entire egg length (Lynch et al. 2010b; Wilson et al. 2011). In *Nasonia*, functional studies show that EGF signaling negatively regulates the formation of eggshell cues required to localize a ventral source for embryonic DV patterning (Lynch et al. 2010b; Özüak et al. 2014). However, both hymenopteran species are also able to localize the RNA for BMP signaling components along the dorsal midline of the oocyte.

For *Apis*, dorsal localization has been observed for the RNA of the ligand Dpp (Wilson et al. 2011) while in *Nasonia*, as shown in this study, the RNA of the type I receptor Tkv is dorsally localized (Fig. 2g, h). If proteins produced by these localized RNAs are inserted into the membrane and remain there until egg deposition and early embryogenesis, they might become sources for establishing signaling gradients in the embryo. In this context, our finding is interesting that one of the type II receptors *Nv-punt1* is expressed along the dorsal midline in blastoderm embryos (Fig. 3e). This pattern might result from a positive feedback mechanism by which BMP signaling initiates local *Nv-punt1* expression that in turn potentiates BMP signaling. The broad BMP signaling gradient we detected by pMAD staining in early embryos (Özüak et al. 2014) might result from the maternal localization of signaling components in the oocyte such as *Nv-tkv* RNA. The later refinement of the gradient, which leads to high levels of BMP signaling along the dorsal midline, might reflect the positive feedback involving *Nv-punt1*. Further studies are required to investigate whether maternal receptor localization coupled to zygotic positive feedback can account for the long-rang effects of BMP signaling in *Nasonia* which in contrast to *Drosophila* and *Tribolium*, are responsible for patterning the entire DV axis of the embryo (Özüak et al. 2014).

## Electronic supplementary material

Below is the link to the electronic supplementary material.

Supplementary Figure 1 Part of a Smad protein alignment showing the conserved MH2 and SXS domain. (PNG 643 kb)

ESM 1 (DOCX 155 kb)

ESM 2 (DOCX 107 kb)
