# Supplementary figures and images for: Ancient and diverged TGF-β signaling components in Nasonia vitripennis
Source: Dev Genes Evol. 2014 Oct 11;224(4):223–33. doi: 10.1007/s00427-014-0481-0 (PMC4218986; doi:10.1007/s00427-014-0481-0)

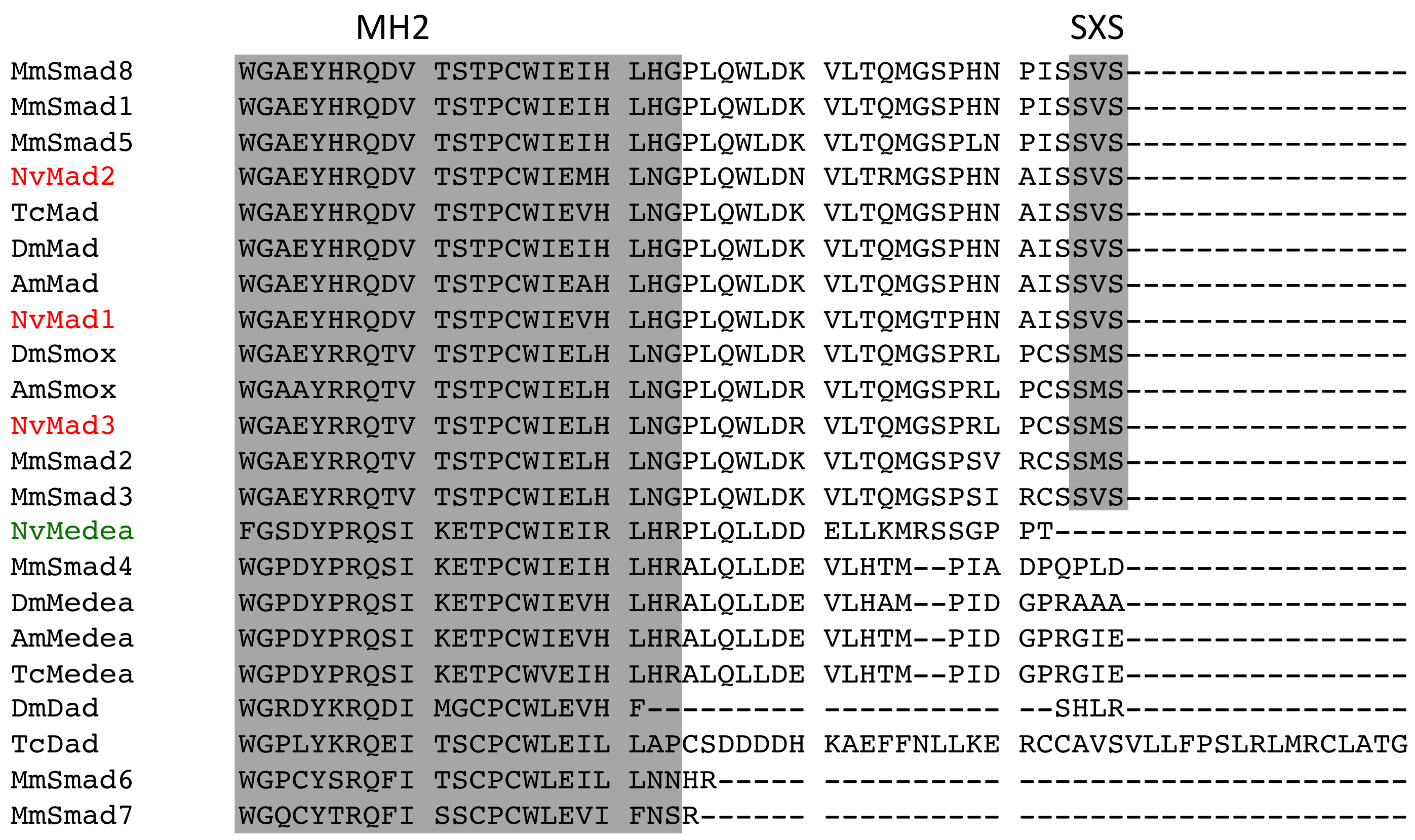

Supplement: Supplementary file 1 — Part of a Smad protein alignment showing the conserved MH2 and SXS domain. (PNG 643 kb) [file 427_2014_481_MOESM1_ESM.png]
