# Supplementary material for: Ancient and diverged TGF-β signaling components in Nasonia vitripennis
Source: Dev Genes Evol. 2014 Oct 11;224(4):223–33. doi: 10.1007/s00427-014-0481-0 (PMC4218986; doi:10.1007/s00427-014-0481-0)
Supplement: Supplementary file 2 — (DOCX 155 kb) [file 427_2014_481_MOESM2_ESM.docx]

**ISH Primers**

| Gene | Primer | Sequence | Accession Nr. |
| --- | --- | --- | --- |
| *twist* | Forward | ggCCgCgggCTTCTCgCCCAgTAACAAC | XM_001605767 |
|  | Reverse | CCCggggCACgTTAgCCATgACCCTCTg | XM_001605767 |
| *vnd* | Forward | ggCCgCgggTCggACTgCTCAACAACTg | XM_001604500 |
|  | Reverse | CCCggggCAggTTCCAggAgCTTCgACT | XM_001604500 |
| *dpp* | Forward | ggccgcggGTGGTGGGCGAGGCGGTAAA | XP_001607677.1 |
|  | Reverse | cccggggcCACGACCTTGTTCTCCTCGT | XP_001607677.1 |
| *gbb1* | Forward | ggccgcggCCAAGTTCCTCCTGGACATC | XP_001603876.1 |
|  | Reverse | cccggggcGATGATCCAGTCCTGCCACT | XP_001603876.1 |
| *gbb2* | Forward | ggccgcggATCCTGCTGCAGTTCGACTT | XP_001603269.2 |
|  | Reverse | cccggggcCCCTGATGATCATGTTGTGG | XP_001603269.2 |
| *activin* | Forward | ggccgcggGACGACTTCTACGCGAGGAC | XP_001602284.1 |
|  | Reverse | cccggggcCTCGATCACGTGCGTGTAGT | XP_001602284.1 |
| *alp* | Forward | ggccgcggAGCTTCTACGGCAAGACCAA | XP_003425497.1 |
|  | Reverse | cccggggcGAGCAGCAGGGCACTATTTC | XP_003425497.1 |
| *myostatin* | Forward | ggccgcggCTCTCGCTTTGGATCTACGG | XP_001602255.2 |
|  | Reverse | cccggggcCGATCTGGTACTCGTTGTCG | XP_001602255.2 |
| *maverick* | Forward | ggccgcggGGCGAGTCAAAGAAGTGCTG | XP_001606148.2 |
|  | Reverse | cccggggcTCAGGAGCAAGCGCACTC | XP_001606148.2 |
| *mad1* | Forward | ggccgcggGCCCCACAACGTCTCATACT | XP_001601460.2 |
|  | Reverse | cccggggcACATCCTGGCGATGGTACTC | XP_001601460.2 |
| *mad2* | Forward | ggccgcggTGAATCAACCATGCCTCAAA | XP_001602991.1 |
|  | Reverse | cccggggcGCATCTCGATCCAACAAGGT | XP_001602991.1 |
| *mad3* | Forward | ggccgcggCAGAAGAGGGACGAGGTCTG | XP_001608214.2 |
|  | Reverse | cccggggcTGTCGTCTTGTTTGCTCGAC | XP_001608214.2 |
| *tkv* | Forward | ggCCgCggTACCCCACATCCAggAgAAg | XP_001601240.2 |
|  | Reverse | CCCggggCgAAgATCTCggTgTgCAggT | XP_001601240.2 |
| *sax* | Forward | ggCCgCgggCTCTTACCCTCCAgACACg | XP_003426889.1 |
|  | Reverse | CCCggggCTgCTTTTgTgTgCCAACATT | XP_003426889.1 |
| *baboon* | Forward | ggccgcggTGCGAAACTGATGGCTACTG | XP_003427942.1 |
|  | Reverse | cccggggcTCCGACGATCTCCATGTGTA | XP_003427942.1 |
| *puntI* | Forward | ggccgcggTCTCAAGCAGAGCAGGGAAT | XP_001606053.1 |
|  | Reverse | cccggggcGAAGCAAGTTCCCAGAGCAC | XP_001606053.1 |
| *puntII* | Forward | ggccgcggGCGCCTACCTAGCGATACTG | XP_001603863.1 |
|  | Reverse | cccggggcCTTCTCAACGCCGATAAAGC | XP_001603863.1 |
| *follistatin* | Forward | ggCCgCggAgCgAggAggACTACgACAA | XP_001607105.2 |
|  | Reverse | CCCggggCAgCTCggATAggTgACgTTg | XP_001607105.2 |
| *cv2a* | Forward | ggccgcggGGCGTTATTACGGAATCGAA | XP_001601040.2 |
|  | Reverse | cccggggcTCGCCGAAGACAGTACACAC | XP_001601040.2 |
| *cv2c* | Forward | ggccgcggGTTGTCCTCATTGCGAAGGT | XP_001603432.2 |
|  | Reverse | cccggggcTCGGTATGGCAAATCAACAA | XP_001603432.2 |
| *cv2d* | Forward | ggccgcggAGCGTCACTGCACAACTGTC | XP_001599102.2 |
|  | Reverse | cccggggcCAAGTTGTCTGCTGCTCTCG | XP_001599102.2 |
| *dally* | Forward | ggccgcggGCGACATTCCCAAACAGATT | XP_003425386.1 |
|  | Reverse | cccggggcGACAACCGTCTTGGCGTATT | XP_003425386.1 |
| *glypican4* | Forward | ggccgcggGCACACAAGGGCCACTAAAT | XP_001607767.2 |
|  | Reverse | cccggggcCGTCGTCGATCTCTTTGTGA | XP_001607767.2 |
| *collagenIV* | Forward | ggccgcggCTTCGGAACGTGGAGAGAAG | XP_003427100.1 |
|  | Reverse | cccggggcGCCAACCAGAAGCTGAACTC | XP_003427100.1 |
| *pentagone* | Forward | ggccgcggCCTAGACCGGGACGAGTACA | XP_001601094.2 |
|  | Reverse | cccggggcGTACCTCGGCAGTTTCTTGC | XP_001601094.2 |
