# Supplementary material for: Ancient and diverged TGF-β signaling components in Nasonia vitripennis
Source: Dev Genes Evol. 2014 Oct 11;224(4):223–33. doi: 10.1007/s00427-014-0481-0 (PMC4218986; doi:10.1007/s00427-014-0481-0)
Supplement: Supplementary file 3 — (DOCX 107 kb) [file 427_2014_481_MOESM3_ESM.docx]

**NCBI accession numbers for ligands**

Am Dpp XP_001122815.2; Dm Dpp AAN10431.1; Tc Dpp EFA02913.1; Mm BMP2 NP_031579.2; Mm BMP4 AAC37698.1; Mm BMP10 NP_033886.2; Mm BMP9 AAD56961.1; Mm GDF5 NP_032135.2; Mm GDF6 NP_038554.1; Mm GDF7 NP_038555.1; Dm Scw AAN11056.2; Dm Gbb AAF47075.1; Am Gbb XP_394252.1; Tc Gbb1 EFA04645.1; Tc Gbb2 EFA04646.1; Mm BMP5 NP_031581.2; Mm BMP8 NP_001242948.1; Mm BMP6 NP_031582.1; Mm BMP7 NP_031583.2; Mm BMP3 NP_775580.1; Mm BMP3B NP_665684.2; Am ADMP XP_003251013.1; Gg ADMP AAD52011.1; Dr ADMP NP_571951.2; Tc Mav EFA11885.1; Dm Mav AAF59328.1; Am Mav XP_001122118.2; Am Alp XP_001122210.1; Dm Alp NP_523461.1; Tc Alp EFA11884.1; Am Act XP_001123044.2; Dm Act NP_651942.2; Tc Act EFA05602.1; Mm Act NP_032408.2; Mm InhbA NP_032406.1; Mm InhbB NP_032407.1; Mm InhbC NP_034695.1; Dm Myo AAF59319.1; Tc Myo EFA05753.1; Mm Myo AAO46885.1; Mm GDF11 NP_034402.1; Mm TGF1 NP_035707.1; Mm TGF2 NP_033393.2; Mm TGF3 NP_033394.2; Nv Dpp XP_001607677.1; Nv Gbb1 XP_001603876.1; Nv Gbb2 XP_001603269.2; Nv ADMP XP_001604750.2; Nv Myo XP_001602255.2; Nv Mav XP_001606148.2; Nv Act XP_001602284.1; Nv Alp XP_003425497.1

**NCBI accession numbers for receptors**

Tc Tkv EFA09250.1; Dm Tkv AAN10533; Am Tkv XP_391989; Mm BMPRIA P36895; Mm BMPRIB NP_031586; Tc Sax EFA07576.1; Dm Sax AAF59189; Am Sax XP_001121528; Mm ActRI NP_031420; Mm ActRIB NP_031421; Mm ALK1 Q61288; Tc Babo EFA01312.1; Dm Babo AAF59011; Mm TGFbrI XM_006537756.1; Mm ALK7 NP_620790; Tc Wit XP_974821.1; Am Wit XP_397334; Dm Wit AAF47832; Mm BMPRII NP_031587; Tc Put EEZ97734.1; Am ActR XP_395928; Dm Put AAF55079; Mm ActRII CAM14875; Mm ActRIIB NP_031423; Nv Wit XP_003428148.1; Nv Tkv XP_001601240.2; Nv Sax XP_003426889.1; Nv Babo XP_003427942.1; Nv Put1 XP_001606053.1; Nv Put2 XP_001603863.1;

**NCBI accession numbers for SMADs**

Dm Mad AAF51142.1; Dm Smox NP_511079.1; Am Mad XP_392819.3; Am Smox XP_396056.4; Mm Smad1 NP_032565.2; Mm Smad5 NP_032567.1; Mm Smad8 AAF77079.2; Mm Smad2 NP_034884.2; Mm Smad3 NP_058049.3; Dm Dad NP_477260.1; Tc Dad EEZ99343.1; Mm Smad6 NP_032568.3; Mm Smad7 NP_001036125.1; Am Medea XP_392838.4; Tc Medea EFA11586.1; Dm Medea NP_524610.1; Mm Smad4 NP_032566.2; Nv Mad1 XP_001601460.2; Nv Mad2 XP_001602991.1; Nv Mad3 XP_001608214.2; Nv Medea XP_003427724.1; Tc Mad EFA05663.1

**NCBI accession numbers for Crossveinless2**

Nv Cv2a XP_001601040.2; Nv Cv2b XP_001599339.1; Nv Cv2c XP_001603432.2; Nv Cv2d XP_001599102.2; Dm Cv2 AAG01337.2; Tc Cv2 EFA10783.1; Am XM_006570432.1; Gg NP_001007081.1; Mm Cv2 AAN45857.1; Xl Cv2 AAX12852.1; Hs Cv2 AAP89012.1;
